# Efficient capture of circulating tumor cells with low molecular weight folate receptor-specific ligands

**DOI:** 10.1038/s41598-022-12118-3

**Published:** 2022-05-20

**Authors:** Yingwen Hu, Danyang Chen, John V. Napoleon, Madduri Srinivasarao, Sunil Singhal, Cagri A. Savran, Philip S. Low

**Affiliations:** 1 Department of Chemistry, Purdue Center for Cancer Research, Purdue Institute for Drug Discovery, Purdue University, 720 Clinic Drive, West Lafayette, IN 47907 USA; 2 Division of Thoracic Surgery, Department of Surgery, Perelman School of Medicine, University of Pennsylvania, Philadelphia, PA 19104 USA; 3 School of Mechanical Engineering, Birck Nanotechnology Center, Purdue Center for Cancer Research, Purdue University, 1205 W. State St., West Lafayette, IN 47907 USA

**Keywords:** Cancer, Chemistry

## Abstract

Retrieval of circulating tumor cells (CTC) has proven valuable for assessing a patient's cancer burden, evaluating response to therapy, and analyzing which drug might treat a cancer best. Although most isolation methods retrieve CTCs based on size, shape, or capture by tumor-specific antibodies, we explore here the use of small molecule tumor-specific ligands linked to magnetic beads for CTC capture. We have designed folic acid-biotin conjugates with different linkers for the capture of folate receptor (FR) + tumor cells spiked into whole blood, and application of the same technology to isolate FR + CTCs from the peripheral blood of both tumor-bearing mice and non-small cell lung patients. We demonstrate that folic acid linked via a rigid linker to a flexible PEG spacer that is in turn tethered to a magnetic bead enables optimal CTC retrieval, reaching nearly 100% capture when 100 cancer cells are spiked into 1 mL of aqueous buffer and ~ 90% capture when the same quantity of cells is diluted into whole blood. In a live animal model, the same methodology is shown to efficiently retrieve CTCs from tumor-bearing mice, yielding cancer cell counts that are proportional to total tumor burden. More importantly, the same method is shown to collect ~ 29 CTCs/8 mL peripheral blood from patients with non-small cell lung cancer. Since the ligand-presentation strategy optimized here should also prove useful in targeting other nanoparticles to other cells, the methods described below should have general applicability in the design of nanoparticles for cell-specific targeting.

## Introduction

Cancer cell metastases accounts for ~ 90% of all cancer deaths^1^. The presence of circulating tumor cells (CTCs) in the bloodstream constitutes an essential intermediate in many metastatic processes and is consequently associated with poor prognosis^2–5^. Detection, quantitation and analysis of CTCs has been shown to provide information on the possible presence of malignant lesions, their driver mutations, their responses to therapy, and their probabilities of recurrence, arguing that development of sensitive methods for the capture and isolation of CTCs could become a useful tool for guiding patient management^6–10^. The rarity of CTCs (often < 10 CTCs per mL patient blood), however, requires that an efficient method be developed for harvesting CTCs from whole blood samples^11,12^.

Initial methods for isolating CTCs from peripheral blood have relied on the binding of CTCs to magnetic beads derivatized with antibodies to epithelial cell-specific markers such as EpCAM or one of the prominent cytokeratins^13^. While a significant quantity of useful data has emerged from these techniques, their inabilities to retrieve nonepithelial-derived cancer cells and epithelial-derived cancer cells that have undergone epithelial to mesenchymal transition (EMT) have prompted pursuit of more comprehensive strategies for CTC isolation^14–19^. Orthogonal methods that have exploited size^20^ and/or rigidity^21^ differences between normal cells and malignant cells have more recently been introduced, and although they have found successful applications, they have also been frequently observed to be compromised by contamination with white cells^20,21^. When antibody-based methods were subsequently expanded to enable capture of cancer cells with established tumor markers such as EGFR^22–24^, EPHB4^25^, erbB-2^26,27^, CEA^23,28^, and Muc-1^29–31^, an ability to retrieve nearly all cancer cells from a blood sample was finally achieved. However, the associated costs and manufacturing constraints have limited commercialization of these tools^32^.

In an effort to produce a highly effective, physically stable, easily manufactured, and inexpensive CTC capture bead capable of isolating the vast majority of human CTCs, we undertook to examine whether low molecular weight organic ligands might be substituted for antibodies for collection of CTCs from peripheral blood. Although many tumor-specific organic ligands could have been explored in this initial study^33^, we elected to test folic acid first, because (1) a receptor for folic acid is expressed on > 40% of all human cancer cells^34^, (2) the folate receptor (FR) is rarely expressed on normal cells^35^, (3) folate binds FR with antibody-like (subnanomolar) affinity^36^, (4) nanoparticles derivatized with folic acid have been repeatedly shown to mediate their binding to cancer cells^37–41^, and (5) many other organic ligands of similar size, tumor specificity, and affinity have been developed that could be combined with folate to create a pan-cancer capture bead if folate-mediated capture were to prove successful^42–48^.

In the paper below we describe: (i) the design of various folic acid-biotin conjugates for use in anchoring folic acid onto streptavidin-coated magnetic beads, (ii) characterization of an optimal spacer for bridging the biotin to folic acid, (iii) use of the resulting folate-linked magnetic bead to capture of FR^+^ tumor cells spiked into whole blood, and (iv) application of the same technology to isolate FR^+^ CTCs from the peripheral blood of both tumor-bearing mice and non-small cell lung patients.

## Methods

Streptavidin coated magnetic beads were purchased from Bangs Laboratories (catalogue-CM01N). Dynabeads MyOne Streptavidin T1 beads were purchased from Invitrogen (catalogue-65601). Biotin-labeled anti-human EpCAM antibody was obtained from BioLegend (Catalog #324,215). Cell culture medium RPMI-1640, CellTracker CM-DiI Dye and Dynal-MPC-L magnetic particle concentrator were obtained from Life Technologies (Grand Island, NY). Fluorenylmethyloxycarbonyl (fmoc) protected amino acids and reagents were purchased from various sources. Fmoc-N-amido-dPEG_n_-acid was obtained from Quanta BioDesign, Ltd (Plain City, OH). Fmoc-glu(Otbu)-OH, Fmoc-proline-OH, and Kaiser Test kit were purchased from AnaSpec (Fremont, CA). *N10*-(trifluoroacetyl)pteroic acid was a generous gift from Endocyte Inc. (West Lafayette, IN). All other chemicals were purchased from Sigma Chemical Co. (St. Louis, MO). KB (an FR + HeLa-derived human cervical cancer line) and MDA-MB-231 cells were purchased from ATCC. Folate-FITC was generous gift from On Target Laboratories Inc. (West Lafayette, IN). Non-small cell lung cancer patient blood samples were provided by Dr. Sunil Singhal, University of Pennsylvania. All animal and human tissue-related experiments were performed in accordance with relevant guidelines and regulations. The studies were carried out in compliance with the ARRIVE guidelines.

### Formation of ligand-coated magnetic beads

The detailed synthetic procedures are provided in the “supplementary file”. For preparation of folate-coated magnetic beads, streptavidin coated magnetic beads were gently vortexed for 1 min to promote full disaggregation and then rinsed three times 20 × volume of phosphate-buffered saline (PBS). Beads (50 µg) were then transferred to a 1.5 mL Eppendorf tube and incubated for 1 h at r.t. on a rotator with the desired concentration of folate-biotin conjugate. Unbound folate-biotin was then removed by washing three times with 20 × volume of PBS and beads were stored at 4 °C until use. To prepare the anti-EpCAM-coated streptavidin magnetic beads, streptavidin T1 beads (25 µg) were gently vortexed for 1 min to promote full disaggregation and then rinsed three times 20 × volume of PBS. Beads were then transferred to a 1.5 mL Eppendorf tube and incubated for 1 h at r.t. on a rotator with anti-EpCAM antibody (10 mg/mL). This was then washed with 20 × volume of PBS (3 times) and immediately used for the experiment.

### Evaluation of folate-biotin binding to cancer cells in culture

Human KB cells and MDA-MB-231 cells were cultured by sub-confluent passage in folic acid deficient RPMI-1640 medium containing 10% fetal bovine serum (FBS) and 1% penicillin/streptomycin. Cells were incubated with the folate-biotin conjugate for 30 min on ice prior to rinsing three times with 20 × volume of PBS. Folate-biotin derivatized cells were then incubated with FITC-streptavidin for 30 min on ice. 7-AAD was used as live/dead cell marker. Flow cytometry was performed on Accuri C6 (BD) and Accuri C6 software was used for data acquisition and analyses.

### Evaluation of cell recovery efficiency in vitro

A known number of KB or MDA-MB-231 cells were spiked into either culture medium or whole human blood collected from healthy donors under a Purdue University IRB approved protocol. A written informed consent was obtained from all the study participants. One milliliter whole blood sample was diluted with PBS (1:1, v/v) before analysis. After incubation with either folate-coated or anti-EpCAM antibody coated beads, the spiked samples were rinsed three times with PBS and the beads were collected using a Dynal-MPC-L magnetic particle concentrator. The captured cells were then transferred to a confocal dish for confocal microscopy (Nikon A1). For purposes of comparison, ~ 85% and 99% of KB sorted EpCAM and folate receptor positive, respectively, by flow cytometry.

### Tumor cell inoculation and blood sample collection

Briefly, subconfluent MDA-MB-231 cells were washed with 20 × volume of PBS, detached by a brief exposure to a 0.25% trypsin, washed in serum-containing medium, and then resuspended at a concentration of ~ 10^7^ cell per mL in cold serum free medium. The cells were kept on ice for 5–20 min until transplanted into athymic nu/nu mice. Mice were anesthetized by inhalation of 2% isoflurane. 100 μL of tumor cell suspension (~ 10^6^ cells) was injected subcutaneously or intravenously through the tail vein. The tumor size was monitored starting 2 weeks after tumor implant. Mouse blood samples (~ 200 μL) were collected from facial vein of mouse once a week into an EDTA coated capillary blood collection tube. Folate-Pro_6_-PEG_12_-biotin was used for mouse CTC sample analysis. The rest of the protocol was the same as that used in the detection of KB cells spiked into blood. Blood sample was diluted with PBS (1:1, v/v) before analysis. After incubation with folate-coated beads, the mouse blood samples were rinsed three times with 400 μL of PBS and separated from the beads using Dynal-MPC-L magnetic particle concentrator and the captured cells were transferred to a confocal dish for image and analysis.

### CTC detection in patient blood samples

Patients with advanced lung cancer were recruited following informed consent for this study under an approved protocol from University of Pennsylvania IRB. A written informed consent was obtained from all the study participants. Blood samples from 6 non-small cell lung cancer patients were collected. A 10-mL blood sample (including anticoagulant) from each patient was collected for examination. Folate-Pro_6_-PEG_12_-biotin ligand was used for patients’ sample analysis. The rest of the protocol was the same as that used in the detection of KB cells spiked into blood.

### Immunofluorescence analysis

Cells captured from patient samples by folate-coated beads were fixed with 4% paraformaldehyde solution and then permeabilized using 0.3% Triton X-100 in PBS at 4 °C overnight. Before staining, cell samples were pre-incubated with 0.2% gelatin blocking solution at r.t. for 1 h. Alexa Fluor 488 anti-cytokeratin pan reactive antibody (final concentration 10 µg/mL), Alexa Fluor 594 anti-CD45 antibody (final concentration 5 µg/mL), and Alexa Fluor 647 anti-CD44 antibody (final concentration 5 µg/mL) were added in blocking solution for 1 h at r.t. After rinsing the samples with 1 mL PBS for three times, cells were counterstained with 300 nM DAPI solution. Cell samples were transferred to a confocal chip and images were taken by confocal microscope.

### Statistical analyses

GraphPad Prism (Graphpad; San Diego, CA) was used for statistical analyses. All figures reported standard deviation values unless otherwise noted. Data were analyzed using one-way ANOVA, followed by post hoc Tukey test.

### Ethics approval and consent to participate

All mice were handled in accordance with Purdue University’s Institutional Animal Care and Use Committee (IACUC) policies on animal care and human samples were handled as per approved protocols with Institutional Review Board. All methods were performed in accordance with relevant guidelines and regulations.

## Results

### Capture ligand and spacer design

**Figure 1:**
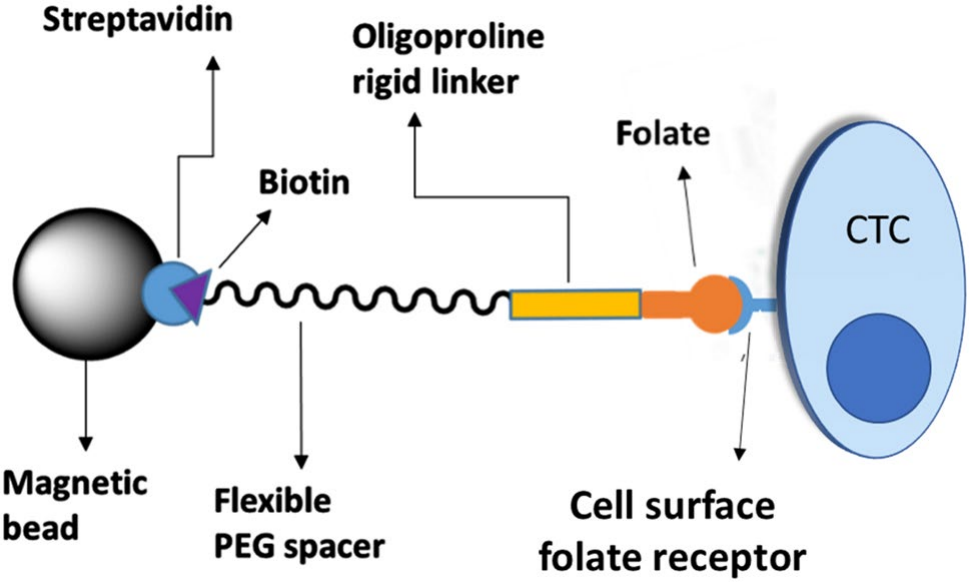
Design of an optimal circulating tumor cell (CTC) capture system.

To date, most CTC capture technologies have relied on tumor-specific antibodies to facilitate CTC binding to an immobilized substrate, nanoparticle or bead^49,50^. In these methodologies, the large size and rigidity of the antibody assures that a substantial fraction of the antigen-binding sites project away from the bead/scaffold surface, thereby orienting the antibody’s binding sites for optimal cancer cell capture. In order to facilitate a similar orientation of a low molecular weight ligand away from a scaffold surface, it seemed prudent to design a spacer that would assure projection of the ligand orthogonal to the surface. For this purpose, we compared various spacers of different lengths and rigidities to determine which spacer chemistry might best enable CTC capture. In all cases, the investigated spacers were conjugated at one end to the capture ligand (e.g. folic acid) and at the other end to the vitamin biotin (Fig. 1)^51,52^. Incubation of the resulting bispecific conjugate with streptavidin-coated magnetic beads then rendered the capture ligand accessible to proximal CTCs. Using this approach, the abilities of various spacers to promote retrieval of a known number of cancer cells from various fluids were quantitated.

### Identification of spacers with optimal capture efficiency

**Figure 2:**
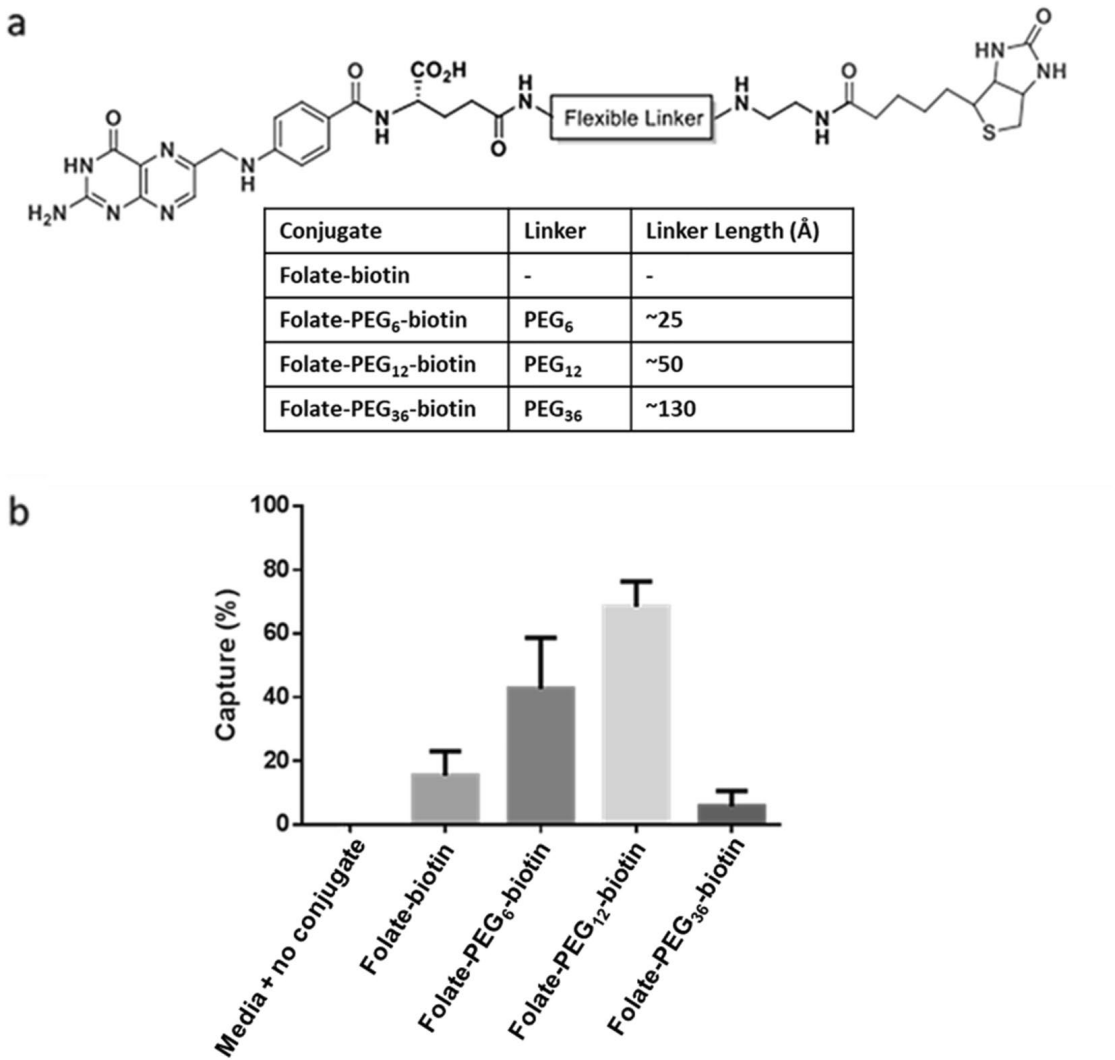
(**a**) Chemical structures of folate-biotin conjugates containing different PEG spacer lengths. (**b**) FR + cell capture efficiency of bispecific folate-biotin conjugate in cell culture media. For analysis of cell capture efficiency, KB cancer cells were spiked into folate-deficient RPMI medium and incubated with folate-conjugated magnetic beads for 1 h. After retrieval of the bead-coated cells with a Dynal-MPC-L magnetic particle concentrator, the captured cells were counted under a microscope.

Because polyethyleneglycol PEG spacers have been frequently employed for ligand presentation^53^, in our initial effort to optimize a small molecule CTC capture strategy, we compared PEG spacers of different lengths (Fig. 2a). As shown in Fig. 2b, the cancer cell capture efficiencies of the bispecific conjugates comprised of PEGs containing 0, 6, 12, and 36 oxyethylene units were highly variable, with the percent of cancer cells retrieved from cell culture medium increasing with spacer lengths from 0 to 12 oxyethylene units and then decreasing precipitously for PEG_36_. This virtual absence of CTC capture by the bispecific tether containing 36 oxyethylene units was surprising in view of the anticipation that longer spacers would facilitate docking of folate to the folate receptors buried deep in the glycocalyx on the cancer cell surface. However, the data argue that ligand accessibility is in fact compromised when too long of a spacer is used, raising the question whether the extraordinary flexibility of the longer PEG spacer might have allowed the tethered folate to fold back towards the bead surface in order to find a thermodynamically more favorable location^54^.

**Figure 3:**
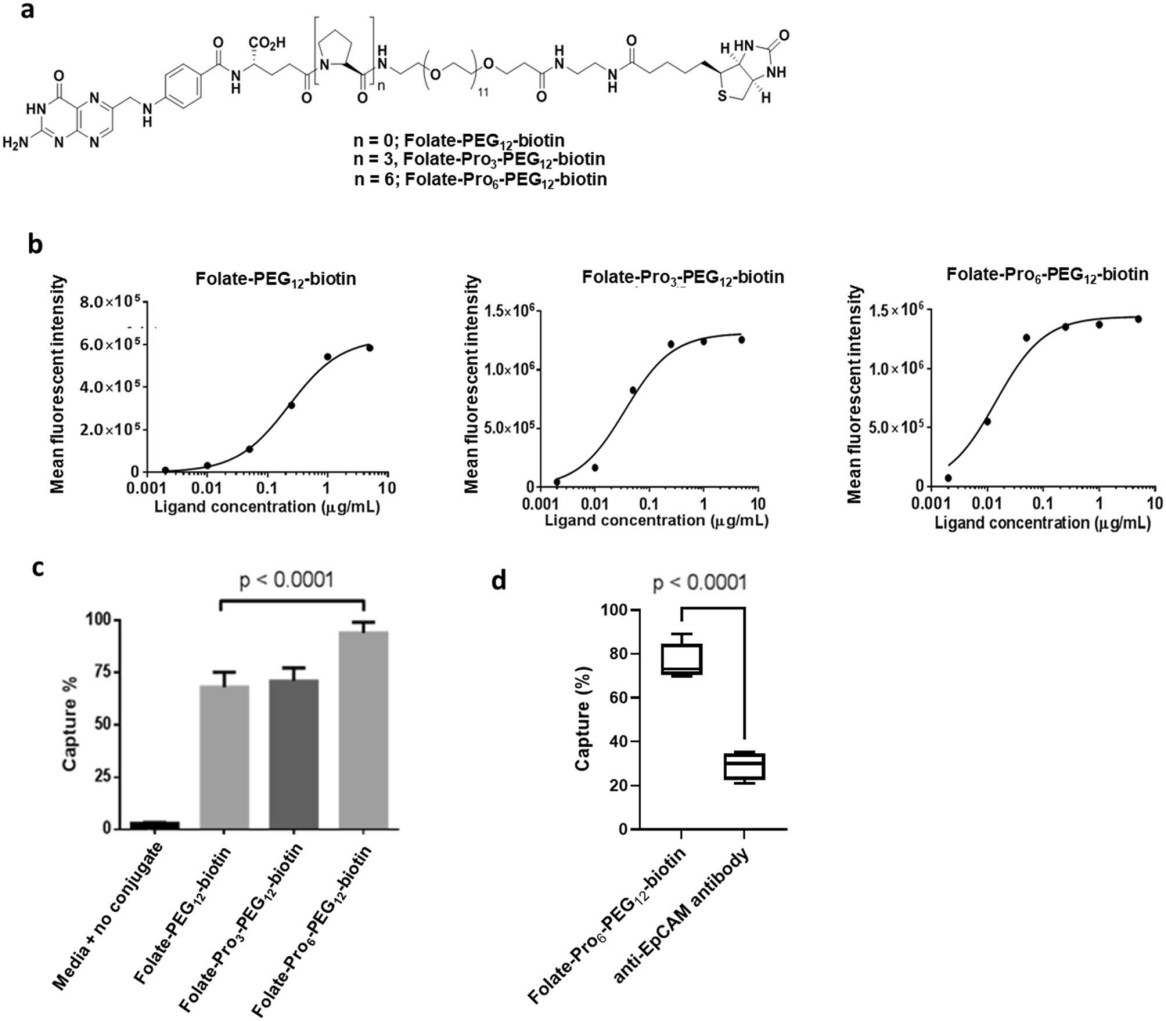
(**a**) Chemical structure of the folate-oligoproline-PEG-biotin conjugates containing a rigid oligoproline linker. (**b**) Binding isotherms of the different folate-biotin conjugates for folate receptor positive KB cells in suspension. (**c**) Comparison of the abilities of the above three folate-biotin conjugates to retrieve KB cancer cells diluted into cell culture medium to a final concentration of 100 cells/mL. (**d**) Comparison of the abilities of folate-Pro_6_-PEG_12_-biotin coated T1 beads with anti-EpCAM coated T1 beads to retrieve KB cancer cells spiked into cell culture medium to a final concentration of 100 cells/mL. Data were analyzed using one-way ANOVA, followed by post hoc Tukey test for the data in panel c and Student’s t test for the data in panel d.

Upon scrutinizing the literature further, we learned that molecular dynamic calculations performed by others^55,56^ had predicted that insertion of a rigid linker between a flexible PEG spacer and a capture ligand should reduce the probability of the ligand burying itself in the PEG “forest” adjacent to a bead surface. To test this prediction, we inserted either 3 or 6 prolines between the folate and the PEG_12_ spacer (Fig. 3a), because oligoprolines are known to form rigid linkers (i.e. proline’s inability to form the two H-bonds necessary to coil into an α-helix causes it to assume a more thermodynamically stable extended rod conformation^57,58^). As shown in the binding analyses of Fig. 3b, the relative avidities for KB cancer cells of beads derivatized with folate-PEG_12_-biotin containing either no proline, a Pro_3_, or a Pro_6_ linker were found to have half-maximal binding concentrations (EC_50_ values) of 0.3, 0.04, and 0.01 µg/mL, respectively, with maximal bead binding levels trending similarly from 6 × 10^5^ to 13 × 10^5^ to 14 × 10^5^ relative units, respectively. Not surprisingly, the abilities of the three bead preparations to retrieve KB cells from culture medium also increased with the same ranking (Fig. 3c). To determine how these optimized folate-derivatized beads might compare with standard anti-EpCAM-based beads, we suspended 100 KB cancer cells in 1 mL of cell culture medium and quantitated the efficiency of cancer cell retrieval using both types of beads. As shown in Fig. 3d, the folate-Pro_6_-PEG_12_-biotin functionalized beads achieved ~ 80% retrieval of the spiked cells, whereas the anti-EpCAM antibody-coated beads captured ~ 30% KB cells. Because the bispecific conjugate with highest binding avidity, Pro_6_, also exhibited the maximum capture efficiency, we elected to conduct all further studies using the folate-Pro_6_-PEG_12_-biotin conjugate.

### Evaluation of the efficiency of CTC capture following addition of CTCs to whole blood

**Figure 4:**
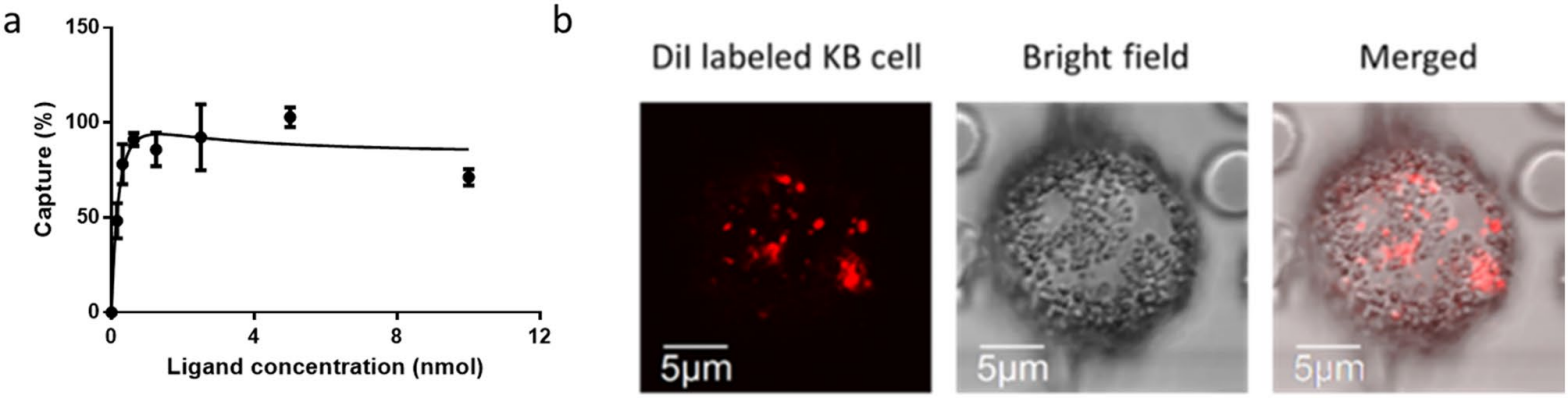
(**a**) Effect of folate-Pro6-PEG12-biotin concentration on the efficiency of capture of spiked FR + KB cells from fresh whole blood obtained from healthy donors. Dynabeads were incubated for 1 h with different concentrations of folate-Pro_6_-PEG_12_-biotin and then washed 3 × in PBS before adding to fresh whole blood spiked with 100 KB cells/mL blood. CTC capture efficiency was then quantitated by counting under microscope (**b**) Fluorescent and white light microscopy image of whole blood smear after incubation with labeled beads showing bead-coated cancer cell surrounded by healthy blood cells. DiI corresponds to 1,1'-Dioctadecyl-3,3,3',3'-Tetramethylindocarbocyanine. Error bars represent standard deviation (n = 3).

Although the aforementioned efficiency of KB cell retrieval from cell culture medium approached 100%, the possibility that serum proteins and blood cells might interfere with CTC capture had not yet been examined. For this purpose, KB cells were suspended in fresh whole human blood at a final concentration of 100 cells/mL and again CTC capture was quantitated, only in this case the bispecific conjugate concentration was also varied in order to determine the optimal folate-biotin density on a bead surface for cancer cell retrieval. As shown in Fig. 4a, maximum cancer cell capture from whole blood (~ 90%) was attained when magnetic beads were preloaded with bispecific conjugate at a concentration of ~ 1.0 nmol/mL, at which point the cancer cell surfaces became nearly saturated with magnetic beads (see Fig. 4b). Moreover, any further increase in conjugate concentration led to no further enhancement of CTC capture, suggesting bead loading at 1.0 nmol/mL likely saturated the beads with folate-Pro_6_-PEG_12_-biotin conjugate.

### Evaluation of CTC capture from blood in tumor-bearing mice

**Figure 5:**
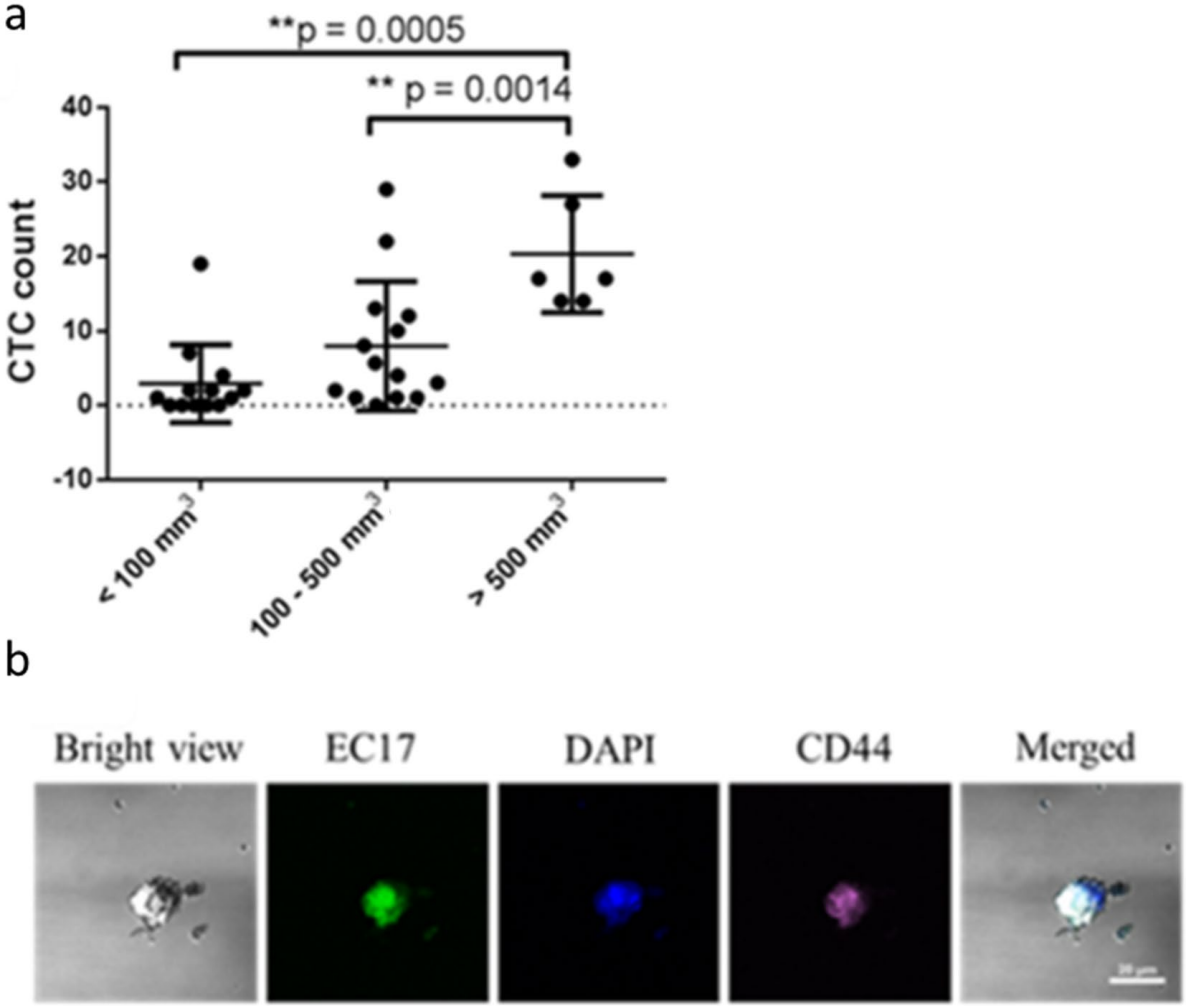
(**a**) Correlations between tumor volume and CTCs captured by folate-biotin coated beads from ~ 200 μL of mouse blood (n > 6). MDA-MB-231 cells were injected subcutaneously into nude mice to build the solid tumor model. Data were analyzed using one-way ANOVA, followed by post hoc Tukey test (**P < 0.0005–0.0014). (**b**) CTCs were captured by folate-biotin coated beads. Captured cells were stained with fluorescently labeled folic acid, folate-FITC (EC17), DAPI or anti-CD44 antibody to label cancer cells, nuclei and stem-like cells, respectively.

Encouraged by the successful capture of cancer cells spiked into whole human blood, we next proceeded to determine whether the same folate-targeted beads might be exploited to capture CTCs from tumor-bearing mice. For this purpose, FR^+^ human breast cancer cells (MDA-MB-231 cells) were implanted subcutaneously in nude mice and allowed to grow to different sizes ranging from < 100 to 1000 mm^3^. Blood was then collected starting at three weeks post tumor cell implantation and analyzed for FR positive and CD44 negative CTCs. As shown in Fig. 5a, the number of endogenous CTCs captured increased with tumor size, with tumors < 100 mm^3^ yielding 3.6 CTCs per 200 µl blood (range 0 – 19, S.D. = 5.5), tumor volumes of 100—500 mm^3^ enabling capture of ~ 9.5 CTCs per 200 µl blood (range from 1 – 29, S.D. = 8.7), and tumors up to 1000 mm^3^ permitting retrieval of ~ 20.3 CTCs (range from 14 – 33, S.D. = 7.1) per 200 µl blood. This correlation between tumor volume and CTCs captured is consistent with previous reports^59^ and suggests that the bead and spacer design explored here can be similarly used to assess tumor growth and response to therapy.

It is also interesting to note that CD44 positive tumor cells are also captured by this technique. While additional markers would have to be analyzed to more accurately characterize these cells, it is interesting to speculate that the CD44 positive cells might be undergoing epithelial to mesenchymal transition (EMT)^60^ (Fig. 5b). This occurs despite the fact that most epithelial markers disappear during EMT^61^, and thereby render EpCAM- and cytokeratin-dependent capture techniques ineffective^19^. This added capability to collect stem-like cancer cells should facilitate analyses of cancer cells that no longer express epithelial markers.

### Evaluation of CTC capture from blood in lung cancer patients

**Figure 6:**
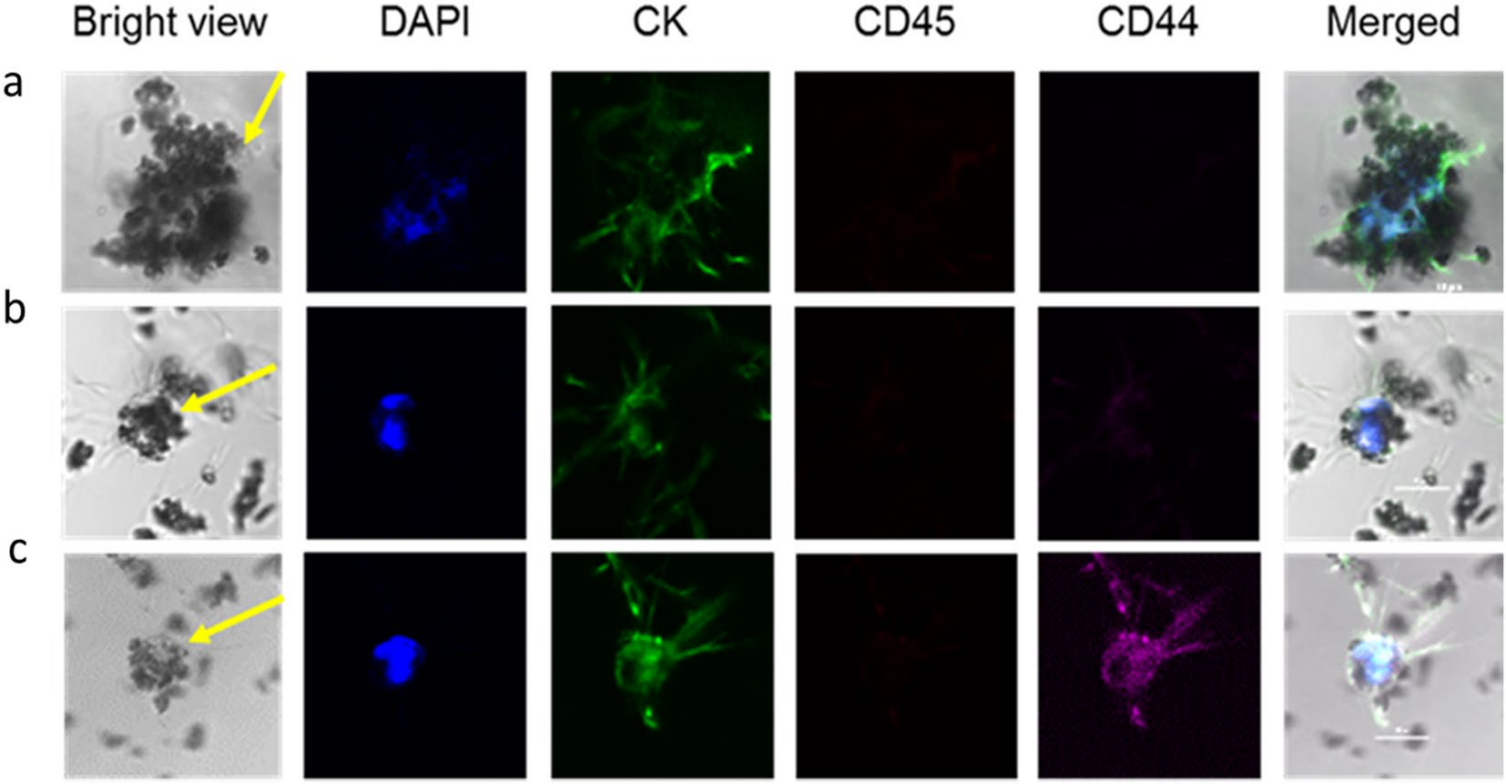
CTC captured and analyzed from lung cancer patient samples. An 8 mL blood sample (including anticoagulant) from each patient was collected for examination. Folate-Pro6-PEG12-biotin ligand-coated beads was used for patient sample analysis. Cell captured ligand-coated beads were stained and analyzed by immunofluorescence. Immunofluorescence staining of cell surface receptors of a captured a) CTC cluster, b) single CTC and c) stem cell like CTC from a lung cancer patient. The images shown staining included Cytokeratin (CK, green), CD45 (red), CD44 (purple), and DAPI (blue). The scale bar is 20 μm.

Finally, to obtain an initial indication of an ability to translate this technology into the clinic, we analyzed blood samples from 6 patients with metastatic lung cancer for FR + CTCs. Cells were identified as CTCs if they stained positive for DAPI (a nuclear stain to prevent counting of erythrocytes and platelets), did not express CD45 (to avoid counting leucocytes), and expressed the epithelial marker, cytokeratin (CK; a marker for epithelial derived CTCs). As shown in Fig. 6a, CTCs could be identified in all six patients samples, with counts ranging from 8 – 84 CTCs per 8 mL sample (mean = 28.8). That these captured cells were indeed CTCs could be confirmed by their examination under the microscope (see representative images in Fig. 6b), where the cells were confirmed to stain positive for cytokeratin and negative for CD45. The fact that a small number of cells (range 0 – 7, mean = 2.7) also stained positive for CD44 confirmed that the beads designed here are indeed capable of capturing CTCs that might be undergoing EMT and display more stem-like properties (Fig. 6c). Perhaps most importantly, the small molecule-derivatized beads created here were also capable of capturing a small number of CTC clusters (range 0 – 14 cells in size, mean = 4.3), which is important since the presence of circulating cell clusters has been associated with poor prognosis and survival^62,63^. Because our bead capture strategy does not employ a size cutoff for CTC selection, single cells, small cell clusters and larger cell clusters could all be easily isolated.

## Discussion

Although the rigid conformation of an antibody generally ensures that at least one of the antibody’s two antigen binding sites will face away from the bead/substrate surface and become available for CTC binding, we have found that flexible PEG spacers do not provide the same benefit. Instead, the flexibility of the PEG spacer enabled the PEG-tethered ligand to fold back toward the bead surface and submerge itself in the surrounding PEG “forest”, thereby render the ligand inaccessible for CTC capture. To reduce this tendency, we were forced to insert a rigid linker (oligoproline) between the PEG tether and the organic ligand. According to molecular dynamics simulations^55,56^, the rigidity of this spacer prevents the organic ligand from avoiding interaction with the aqueous solvent by burying itself in the dense PEG matrix. It is not inconceivable that this simple spacer modification could prove useful in the design of many types of ligand-derivatized nanoparticles.

We have documented here the efficient use of a low molecular weight organic ligand for the selective capture of CTCs. Although antibodies, aptamers, and protein/peptide scaffolds have also proven successful in isolating CTCs^50^, we propose that organic ligands have a few advantages that may prove beneficial. Thus, organic ligands are generally more stable, easier to conjugate to a scaffold or bead surface, more capable of penetrating a dense glycocalyx enshrouding almost all tumor cells, and much cheaper to manufacture.

## Conclusions

Although a preliminary proof of concept for CTC capture was established using folate-derivatized magnetic beads, i.e. a capture ligand whose receptor is expressed on ~ 40% of human cancers but absent or present in very limited numbers on all normal cells except the kidneys^35^, other organic ligands have already been developed for most all other cancers^33,64–68^, and several of these have been shown to readily capture CTCs with an efficiency similar to folate’s (data not shown). Thus, the pathway to creation of a universal magnetic bead with the capability of capturing CTCs from all cancer types would seem to be feasible, i.e. simply derivatize the optimal magnetic bead with a cocktail of bispecific conjugates that can recognize folate receptor negative cancers. Importantly, low molecular weight tumor-specific ligands required for such a broad specificity beads have already been developed for targeting fluorescent dyes and other imaging agents to human cancers^69–74^, including nonepithelial cancers which are not readily detected by many current technologies. Indeed, the ease with which such a broad specificity capture beads could be designed and developed may constitute a major advantage to the low molecular weight ligand strategy described here.

## Supplementary Information

Supplementary Information.

## Data Availability

The datasets used and/or analyzed during the current study are available from the corresponding author on reasonable request.
