# Supplementary material for: Efficient capture of circulating tumor cells with low molecular weight folate receptor-specific ligands
Source: Sci Rep. 2022 May 20;12:8555. doi: 10.1038/s41598-022-12118-3 (PMC9122947; doi:10.1038/s41598-022-12118-3)
Supplement: Supplementary file 1 — Supplementary Information. [file 41598_2022_12118_MOESM1_ESM.docx]

Supplementary File

**General analytical and preparative chromatography procedures**

Preparative reverse-phase high performance liquid chromatography (RP-HPLC) was performed on a Waters, XBridge Prep C18, 5 μm; 19 × 100 mm column, mobile phase A = 20 mM ammonium acetate buffer, pH 5 or 7, B = acetonitrile, system with gradients in 30 min, 13 mL/min, λ = 254/280 nm. The LRMS-ESI (LC-MS) was recorded on Agilent 1220 system, with Waters, XBridge RP18, 3.5 μm; 3 × 50 mm column, mobile phase A = 20 mM ammonium bicarbonate buffer, pH 5 or 7, B = acetonitrile, system with gradients in 12-15 min, 0.75 mL/min, λ = 254/280 nm.

**Synthesis of folate-biotin conjugate**

Commercially available 1,2-diaminoethane trityl resin (30 mg, 1 equiv., 0.051 mmol) was swollen with dichloromethane (DCM) (3 mL) for 15 min followed by isopropyl alcohol (IPA) (2 × 3 mL) for 5 min each time and dimethyl formamide (DMF) (2 × 3 mL) for 10 min each. Then, a solution of Fmoc-glu-(OtBu)-OH (54.3 mg, 2.5 equiv., 0.13 mmol), (Benzotriazol-1-yloxy)tripyrrolidinophosphonium hexafluorophosphate (PyBOP) (66.3 mg, 2.5 equiv., 0.13 mmol), diisopropylethyl amine (DIPEA) (0.089 mL, 10 equiv., 0.51 mmol) in DMF (1.5 mL) was added to the resin and bubbled under nitrogen for 4 h. Then, the resin was again washed with DMF (2 × 3 mL) for 10 min and isopropyl alcohol (IPA) (2 × 3 mL) for 5 min and tested by a Kaiser test.[[49](#_ENREF_49)] After confirming the coupling of glutamic acid on the resin, 20% piperidine in DMF (3 × 3 mL) was added to the resin. Then, the resin was washed with DMF, IPA and confirmed deprotection by a Kaiser test. After swelling the 20 resin in DMF, a solution of *N10*-(trifluoroacetyl)pteroic acid (31.2 mg, 1.5 equiv., 0.0765 mmol), PyBOP (66.3 mg, 2.5 equiv., 0.13 mmol), DIPEA (0.089 mL, 10 equiv., 0.51 mml) in DMF (1.5 mL) was added to the resin and bubbled for overnight. Then, the resin was washed with DMF (2 × 3 mL) for 10 min and IPA (2 × 3 mL) for 5 min. After performing a Kaiser test, the final compound was cleaved from the resin using the cocktail solution (trifuoroacetic acid (TFA): H_2_O: triisopropylsilane (TIPS)) (95:2.5:2.5) (3 × 3 mL) for 30 min each time. The compound was concentrated under vacuum. Excess TFA was evaporated with rotary evaporator. Then, the crude product was precipitated in cold diethyl ether solution and centrifuged at 2000 rpm for 10 min. The supernatant was removed. The product was dissolved in dimethyl sulfoxide (DMSO) and purified on RP-HPLC using a gradient mobile phase of A = 20 mM ammonium acetate buffer (pH = 7) and B = acetonitrile; solvent gradient 0% B to 50% B over 35 min (column: Waters xTerra C18, 10 μm; 19 x 250 mm). Elution of the bispecific conjugates were monitored at λ = 280 nm, 360 nm and the identities of the eluted compounds were analyzed by LC-MS. After the purification, (+)-Biotin N-hydroxysuccinimide ester (1 equiv.) was coupled to the product (1 equiv.) and DIPEA (10 equiv.) in DMSO for 1 h at room temperature (r.t.) under nitrogen. And then the tert-butyl ester which protected carboxyl group on glutamic acid was removed by using hydrogen chloride solution (4.0 M in dioxane) for 1 h at r.t. Finally, the N10-trifluoroacetic acid group was deprotected by using mild 0.5 M ammonia hydroxide (NH_4_OH) buffer for 2 h at r.t. The final compound was purified again by using RP-HPLC in the same condition as above. Characterization of folate-biotin: C_31_H_39_N_11_O_7_S, expected 709, found 710.3; UV/Vis: λmax = 280 nm, 360 nm.

**Synthesis of folate-PEG_n_-biotin conjugates**

1,2-Diaminoethane trityl resin was swollen as described above. Then, a solution of Fmoc-N-amido- dPEG_n_-acid (0.051 mmol, 1 equiv.), PyBOP (66.3 mg, 2.5 equiv., 0.13 mmol), DIPEA (0.089 mL, 10 equiv., 0.51 mmol) in DMF (1.5 mL) was added. After bubbling under nitrogen overnight, the resin was washed with DMF (2 × 3 mL) for 10 min each and IPA (2 × 3 mL) for 5 min each. Then, a Kaiser test was performed and showed negative result. After swelling the resin in DMF, 20% piperidine in DMF (3 × 3 mL) was added to the resin and bubbled under nitrogen for 10 min each time to deprotect the Fmoc group. The resin was then washed with DMF and IPA followed by doing a Kaiser test. A positive result from the Kaiser test was confirmed. After that, a solution of Fmoc-glu-(OtBu)-OH (54.3 mg, 2.5 equiv., 0.13 mmols), PyBOP (66.3 mg, 2.5 equiv., 0.13 mmol), DIPEA (0.089 mL, 10 equiv., 0.51 mmol) in DMF (1.5 mL) was added to the resin and bubbled under nitrogen for 4 h. Then, the resin was again washed with DMF (2 × 3 mL) for 10 min and IPA (2 × 3 mL) for 5 min and tested by a Kaiser test. After confirming the coupling of glutamic acid on the resin, 20% piperidine in DMF (3 × 3 mL) was added to the resin. Then, the resin was washed with DMF, IPA and confirmed deprotection by a Kaiser test. After swelling the resin in DMF, a solution of *N10*-(trifluoroacetyl)pteroic acid (31.2 mg, 1.5 equiv., 0.0765 mmol), PyBOP (66.3 mg, 2.5 equiv., 0.13 mmol), DIPEA (0.089 mL, 10 equiv., 0.51 mml) in DMF (1.5 mL) was added to the resin and bubbled for overnight. Then, the resin was washed with DMF (2 × 3 mL) for 10 min and IPA (2 × 3 mL) for 5 min. After performing a Kaiser test, the final compound was cleaved from the resin as per described in the method above. The compound was concentrated under vacuum. Excess TFA was evaporated with rotary evaporator. Then, the crude product was precipitated in cold diethyl ether solution and centrifuged at 2000 rpm for 10 min. The supernatant was removed. The product was dissolved in DMSO and purified on RP-HPLC using a gradient mobile phase of A = 20 mM ammonium acetate buffer (pH = 7) and B = acetonitrile; solvent gradient 0% B to 50% B over 35 min (column: Waters xTerra C18, 10 μm; 19 x 250 mm). Elution of the bispecific conjugates were monitored at λ = 280 nm, 360 nm and the identities of the eluted compounds were analyzed by LC-MS. After the purification, biotinylation was performed as described above. Characterization of Folate-PEG_n_-biotin: Folate-PEG_6_-Biotin: C_46_H_68_N_12_O_14_S, expected 1045.18, found 1046.4; Folate-PEG_12_-Biotin: C_58_H_92_N_12_O_2_0S, expected 1309.50, found 1309.5; Folate-PEG_36_-Biotin: C_106_H_188_N_12_O_44_S, expected 2368, found 2368; For all the compounds, UV/Vis: λmax = 280 nm, 360 nm.

**Synthesis of folate-Pro_n_-PEG_12_-biotin conjugates**

1,2-diaminoethane trityl resin was swollen as described above. Then, a solution of Fmoc-N-amido- dPEG_12_-acid (0.051 mmol, 1 equiv.), PyBOP (66.3 mg, 2.5 equiv., 0.13 mmol), DIPEA (0.089 mL, 10 equiv., 0.51 mmol) in DMF (1.5 mL) was added. After bubbling under nitrogen overnight, the resin was washed with DMF (2 × 3 mL) for 10 min each and IPA (2 × 3 mL) for 5 min each. Then, a Kaiser test was performed and showed negative result. After swelling the resin in DMF, 20% piperidine in DMF (3 × 3 mL) was added to the resin and bubbled under nitrogen for 10 min each time to deprotect the Fmoc group. The resin was then washed with DMF and IPA followed by doing a Kaiser test. After deprotection of Fmoc group was confirmed by a Kaiser test, a solution of Fmoc-proline-OH (86 mg, 5 equiv., 0.26 mmol), PyBOP (66.3 mg, 2.5 equiv., 0.13 mmol), HOBT (19.5 mg, 2.5 equiv., 0.13 mmol), DIPEA (0.089 mL, 10 equiv., 0.51 mmol) and DMF (1.5 mL) was added. After 4 h, the resin was washed with DMF (2 × 3 mL) for 10 min each and IPA (2 × 3 mL) for 5 min each. Then, the fmoc group was deprotected with 20% piperidine in DMF (3 × 3 mL) and bubbled under nitrogen for 10 min each time. This proline amino acid addition was repeated either two times (for folate-Pro_3_-PEG_12_-biotin) or 5 times (for folate-Pro_6_-PEG_12_-biotin). After that, a solution of Fmoc-Glu-(OtBu)-OH (54.3 mg, 2.5 equiv., 0.13 mmols), PyBOP (66.3 mg, 2.5 equiv., 0.13 mmol), DIPEA (0.089 mL, 10 equiv., 0.51 mmol) in DMF (1.5 mL) was added to the resin and bubbled under nitrogen for 4 h. Then, the resin was again washed with DMF (2 × 3 mL) for 10 min and IPA (2 × 3 mL) for 5 min and tested by a Kaiser test. After confirming the coupling of glutamic acid on the resin, 20% piperidine in DMF (3 × 3 mL) was added to the resin. Then, the resin was washed with DMF, IPA and confirmed deprotection by a Kaiser test. After swelling the resin in DMF, a solution of *N10*-(trifluoroacetyl)pteroic acid (31.2 mg, 1.5 equiv., 0.0765 mmol), PyBOP (66.3 mg, 2.5 equiv., 0.13 mmol), DIPEA (0.089 mL, 10 43 equiv., 0.51 mmol) in DMF (1.5 mL) was added to the resin and bubbled for overnight. Then, the resin was washed with DMF (2 × 3 mL) for 10 min and IPA (2 × 3 mL) for 5 min. After performing a Kaiser test, the final compound was cleaved from the resin as per described in the method above. The compound was concentrated under vacuum. Excess TFA was evaporated with rotary evaporator. Then, the crude product was precipitated in cold diethyl ether solution and centrifuged at 2000 rpm for 10 min. The supernatant was removed. The product was dissolved in DMSO and purified on RP-HPLC using a gradient mobile phase of A = 20 mM ammonium acetate buffer (pH = 7) and B = acetonitrile; solvent gradient 0% B to 50% B over 35 min (column: Waters xTerra C18, 10 μm; 19 x 250 mm). Elution of the bispecific conjugates was monitored at λ = 280 nm, 360 nm and the identities of the eluted compounds were analyzed by LC-MS. After the purification, biotinylation was performed as described above. Characterization of Folate-Pro_n_-PEG_12_-biotin: Folate-Pro_3_-PEG_12_-biotin: C_73_H_113_N_15_O_23_S, expected 1600.85, found 1602.7 (1/2 mass: 801.0), UV/Vis: λmax = 210 44nm, 280 nm and 360 nm; Folate-Pro_6_-PEG_12_-biotin: C_88_H_134_N_18_O_26_S, expected 1892.20, found ½ mass 946.6; UV/Vis: λmax = 210 nm, 280 nm and 360 nm.
